# Ventricular metastasis resulting in disseminated intravascular coagulation

**DOI:** 10.1186/1477-7819-3-29

**Published:** 2005-05-24

**Authors:** Thomas John, Ian D Davis

**Affiliations:** 1 Ludwig Institute Oncology Unit, Austin Health, Heidelberg, Victoria 3084, Australia

## Abstract

**Background:**

Disseminated Intravascular Coagulation (DIC) complicates up to 7% of malignancies, the commonest solid organ association being adenocarcinoma. Transitional Cell Carcinoma (TCC) has rarely been associated with DIC.

**Case presentation:**

A 74-year-old woman with TCC bladder and DIC was found to have a cardiac lesion suspicious for metastatic disease. The DIC improved with infusion of plasma and administration of Vitamin K, however the cardiac lesion was deemed inoperable and chemotherapy inappropriate; given the patients functional status. We postulate that direct activation of the coagulation cascade by the intraventricular metastasis probably triggered the coagulopathy in this patient.

**Conclusion:**

Cardiac metastases should be considered in cancer patients with otherwise unexplained DIC. This may influence treatment choices.

## Background

DIC is characterised by the widespread activation of coagulation. This in turn results in intravascular formation of fibrin and ultimately thrombotic occlusion of small to medium sized vessels [1]. The commonest causes are sepsis and trauma. Malignancy is a well recognised cause of a prothrombotic state, with DIC occurring in up to 7% of solid organ tumours[2]. It is most frequently associated with adenocarcinomas such as pancreatic, breast and prostate cancer. Transitional cell carcinoma is rarely associated with DIC, with very few reports in the literature.

## Case presentation

A 74 year old woman presented with a two day history of haematuria and increasing lethargy one month following palliative radiotherapy to the bladder for a T4 Grade III urothelial carcinoma. Clinical examination revealed large ecchymoses over both upper and lower limbs as well as oral mucosal bleeding without any evidence of petechiae. The patient was apyrexial and vital signs were all within normal limits. There were no other significant findings on cardiovascular, respiratory and abdominal examination.

**Figure 1:**
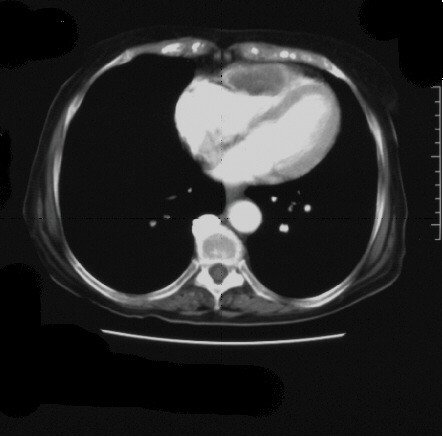
Contrast CT thorax demonstrating filling defect and lesion in right anterior ventricular wall.

**Figure 2:**
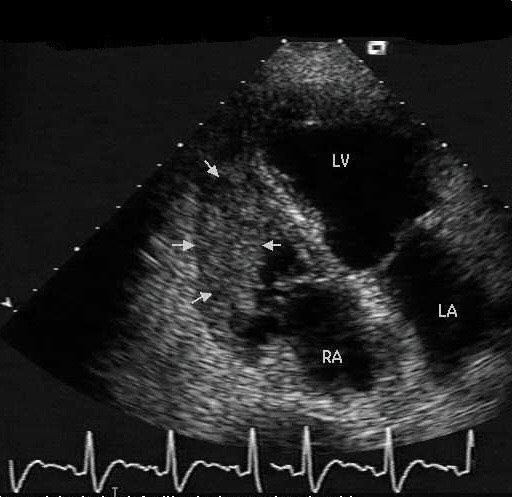
Trans oesophageal echo demonstrating right ventricular mass (arrows) RA: right atrium, LA: left atrium, LV: left ventricle.

Laboratory analysis revealed INR 2.7 (normal range: 0.9–1.3), APTT 73 sec (25–38) D-dimer 25 μg/mL (0–0.2), fibrinogen <0.05 g/L (1.5–4.0), platelets 25 × 10^9^/L (150–400), consistent with disseminated intravascular coagulation (DIC). Computerised tomographic (CT) scan of the thorax and abdomen revealed a lesion within the right ventricle (Figure 1) without other evidence of metastatic disease within the pulmonary or hepatic parenchyma. The primary site appeared necrotic consistent with recent radiotherapy. Trans-oesophageal echocardiography showed that the lesion extended into the body of the right ventricle and significantly reduced ventricular volume (Figure 2). The edges of the lesion were fimbriated and mobile, consistent with a metastatic deposit. Although the platelet count and clotting improved with Vitamin K and plasma, the platelet count did not rise above 50. This was adequate in controlling her haematuria and further treatments were therefore not initiated. As the patient did not show any overt signs of thrombosis, we did not institute therapy with anticoagulants. As the patient's functional status was declining, we were unable to consider chemotherapeutic options. Palliative radiotherapy to the heart was considered but the patient deteriorated and died three months later. A request to perform an autopsy was refused by the patient's family.

## Discussion

Although a hypercoagulable state exists in virtually all patients with malignancy, the incidence of overt DIC is much lower [3]. The aetiology of the activation of coagulability in malignancy is multifactorial and poorly understood. Molecular changes which may influence hypercoagulability include the expression of tissue factor (TF) as well as the proteases hepsin and cancer procoagulant by circulating tumour cells [4].

TF assembles with factor VIIa to activate factors IX and X thereby triggering thrombin formation. TF has been demonstrated to exist in normal vascular endothelial cells suggesting a role in initiating rapid activation of coagulation. It is rarely expressed in normal epithelial tissue but has been found to be expressed in malignant tissue such as breast cancer, whilst not being present in patients with benign fibrocystic disease [5]. The degree of tissue factor expression has been shown to correlate with metastatic potential and histological differentiation such that increased TF expression results an increased potential to metastasise and a more undifferentiated histological appearance [6,7]. Cancer procoagulant has been found in malignant and foetal tissue, but not in normally differentiated tissue[8,9]. Its only known property is the calcium and vitamin K dependent conversion of factor X into Xa, independent of the TF / factor VIIa complex. Hepsin, a protease found on hepatocyte surface membranes but also in several tumour cell lines, has been shown to initiate coagulation by activating factor VIIa independent of TF [10].

The heart is an under-recognised site of metastatic disease in patients with cancer. In one series, the incidence at autopsy was 1.25%. The commonest types of malignancy to spread to the heart in this series were lung and oesophageal cancers as well as lymphomas [11]. This reflects the direct proximity of these tumours to the heart, with direct invasion being the likely pathogenesis. Lung, breast, soft tissue carcinomas, renal cell carcinomas, lymphomas and melanoma are most frequently associated with cardiac involvement. This usually results in pericardial or epicardial disease, with endocardial metastases being less common. There are reports of cardiac metastases from metastatic TCC in the literature, however we could find only one other report of a patient with a cardiac metastases presenting with DIC [12] and this was associated with cervical carcinoma.

The mechanism of DIC in the described case is speculative but may relate to the activation of coagulation pathways directly by the interaction of tumour antigens with plasma [13]. The postulated mechanism of DIC in this case is the direct exposure of tumour cells within the right ventricle to circulating plasma, thus triggering direct activation of the coagulation cascade through mechanisms mentioned above. Although we are unable to prove causality, given that TCC is rarely associated with DIC and that this phenomenon only developed when the tumour metastasised to the patient's heart; this explanation appears more plausible than the assumption that the patients malignancy alone resulted in DIC.

## Conclusion

This case demonstrates that cardiac metastases should be considered in all cancer patients with otherwise unexplained DIC and that this may influence treatment choices.

## Conflict of interest

The author(s) declare that they have no competing interests.

## Authors' contributions

**TJ **– principal author: preparation of manuscript, literature review, acquisition of images.

**ID **– revision of manuscript and figures.

All authors read and approved the final manuscript.
